# Recovery in a Case of Collapse with Vomiting and Diarrhœa, by the Intravenous Injection of Salt Solution

**Published:** 1893-07

**Authors:** 


RECOVERY IN A CASE OF COLLAPSE WITH VOMITING AND DIARRHŒA, BY THE INTRAVENOUS INJECTION OF SALT SOLUTION.—STURGES (*Lancet*, January 9, 1892) reports a case of diarrhœa and vomiting in a child 9 months old. The little patient was collapsed and there was no longer hope of recovery. As a last remedy an intravenous injection of the following mixture was ordered: 12 ounces of distilled water at a temperature of 101° F.; 36 grains of salt; 1 drachm cognac. The result was astonishing. The child reacted in a few hours. The vomiting occurred but once and the diarrhœa ceased. The child made a perfect recovery.—*Ex.*

